# Genome-Wide Association Analysis Identifies Important Haplotypes and Candidate Gene *XKR4* for Body Size Traits in Pekin Ducks

**DOI:** 10.3390/ani14162349

**Published:** 2024-08-14

**Authors:** Jiang-Zhou Yu, Jun Zhou, Fang-Xi Yang, Jin-Ping Hao, Zhuo-Cheng Hou, Feng Zhu

**Affiliations:** 1 National Engineering Laboratory for Animal Breeding, Key Laboratory of Animal Genetics, Breeding and Reproduction of the Ministry of Agriculture, College of Animal Science and Technology, China Agricultural University, Beijing 100193, China; jzyu@cau.edu.cn (J.-Z.Y.); zhoujun138@cau.edu.cn (J.Z.); zchou@cau.edu.cn (Z.-C.H.); 2 Beijing Nankou Duck Breeding Technology Co., Ltd., Beijing 102202, China; yangfangxi@163.com (F.-X.Y.); haojp73@126.com (J.-P.H.)

**Keywords:** GWAS, pekin duck, haplotype, body size

## Abstract

**Simple Summary:**

The body size of ducks reflects their skeletal development and growth status. However, the rapid growth and early large body size of meat ducks negatively affect subsequent slaughter and processing. Therefore, it is necessary to identify candidate genetic markers and genes involved in body size regulation. In this study, we conducted genome-wide association studies (GWASs) on the body length (BL), keel bone length (KBL), neck length (NL), and breast width (BrW) of 42-day-old Pekin ducks (Anas platyrhynchos domestica). The results showed that BL and KBL had similar GWAS signals. Based on these results, we further identified causal haplotypes and candidate genes affecting BL and KBL. This research provides new genetic insights into the growth and development of Pekin ducks and has important implications for their breeding improvement.

**Abstract:**

Body size is an important growth indicator in ducks and is a primary selection criterion for physical improvement. An excessively rapid growth rate in meat ducks can result in excessive body size, which may hinder subsequent processing and slaughter operations. However, only a few molecular markers related to body size have been studied in meat ducks. In this study, we performed a genome-wide association study (GWAS) to identify candidate genes and QTLs affecting body length (BL), keel bone length (KBL), neck length (NL), and breast width (BrW) in Pekin ducks (Anas platyrhynchos domestica). Our results indicate the significant SNP for NL is located within a pseudogene, whereas the significant SNP for BrW is located in an intergenic region. More importantly, our analysis identified a haplotype that was significantly associated with both BL and KBL. This haplotype, containing 48 single-nucleotide polymorphisms (SNPs), is localized within the *XKR4* gene. The identification of this haplotype suggests that *XKR4* may be a key candidate gene influencing BL and KBL in Pekin ducks. These findings have important implications for the breeding and genetic improvement of Pekin ducks, and provide valuable insights into the genetic architecture of body size traits in this species.

## 1. Introduction

Duck meat occupies a significant position in the global meat market, being the third largest meat industry after pork and chicken [1]. Body size is an important indicator of the growth status of animals, and the development of their skeleton and skeletal muscles [2]. Skeletal development is a key factor in weight gain, and studies have shown that bigger size means higher meat production. [3]. In addition, carcasses and other by-products of duck have high added value in food processing. However, excessive size has a negative impact on slaughter and processing. For example, larger ducks can be more difficult to process accurately at various stages such as depilation, evisceration, and cutting, leading to inconsistent meat quality and negative economic effects.

Previous studies have shown that body size in poultry may be regulated by multiple genes. Cao et al. found that exon polymorphisms in *PITX2* significantly affect the body and carcass traits of Wuliang Mountain Black-bone chickens [4]. Hong et al. found that an SNP downstream of *BMP2* can explain nearly 10% of the genetic variation in body length and height in Large White pigs [5]. Compared to chickens and pigs, fewer studies have been conducted on molecular markers related to body size traits in ducks. Zhou et al. constructed F2 hybrid populations of domestic and mallard ducks and found that a haplotype on the IGF2BP1 gene significantly affected body size using genome-wide association analysis (GWAS) [6]. However, this haplotype has been completely fixed in the domestic duck genome over a long period of selection and breeding. In a previous study, we carried out a GWAS of body size and carcass traits in ducks using Genotyping-by-Sequencing (GBS) [7]. Limited by marker density and coverage, a very small proportion of QTLs were detected. Therefore, few effective genomic markers can be used for molecular breeding of Pekin duck body size traits.

In this study, we aim to detect the variation in Pekin ducks by genome resequencing, followed by screening novel functional genes and QTLs related to body size by genome-wide association analysis, haplotype analysis, and other analytical methods. The results of the study can provide candidate genes for subsequent functional studies as well as basic data for the genomic selection of body size traits.

## 2. Materials and Methods

### 2.1. Phenotypes and Genotypes

In this study, we measured BL, KBL, NL, and BrW in 642 Pekin ducks (336 males, 306 females) at 42 days of age. These ducks were from three different batches within the same year and were all provided by Beijing Nankou Duck Breeding Technology Ltd. They were raised under identical environmental and feeding conditions. In particular, they were provided ad libitum commercial diets. They were fed a starter diet from 1 to 18 days of age, which contained 19% crude protein (CP) and 12.81 MJ/kg of dietary metabolizable energy (ME). Following this, they received a grower diet containing 17.1% CP and 11.95 MJ/kg ME until 42 days of age.

The BL, KBL, NL, and BrW were measured using a caliper. BL, the distance between the shoulder joint and the ischial tuberosity; KBL, the distance between the anterior and the posterior border of the breast-bone crest; NL, the distance between the first cervical vertebra and the end of neck; BrW, the distance vertically between the backbone and the beginning of the breast-bone crest.

Blood samples were collected, and whole-genome DNA was extracted from the blood samples using a QIAamp DNA Blood Mini Kit (QIAGEN, Hilden, Germany). The whole-genome sequencing data were generated on the Illumina HiSeq 4000 platform using 150 bp paired-end reads, with an average sequencing depth of 2.11×.

### 2.2. Statistical Analysis

Phenotypes exceeding the Mean ± 3SD range were excluded as outliers. Normality was tested using the Shapiro–Wilk test. If phenotypes deviated from normal distribution, rank transformation [7,8,9] was applied to normalize them for the mixed linear model [10].

Adapters and low-quality reads were removed from all sequencing files using Fastp [11]. The sequencing data were aligned to the reference genome using Sentieon [12], with the reference being the chromosome assembly of the mallard genome (GenBank: ASM874695v1). SNP detection and imputation were performed using STITCH [13], with parameters K = 10 and nGen = 120, keeping other settings default. SNPs with info_score < 0.9 were removed to ensure imputation accuracy. Autosomal SNPs were retained and further filtered using Vcftools with the following criteria: minor allele frequency > 0.01, SNP call rate ≥ 0.95, and individual call rate ≥ 0.8. A total of 4,603,480 SNPs were obtained for GWAS. Principal component analysis (PCA) was conducted by Plink [14] and multiple testing correction for the GWAS was performed using SimpleM [15] to determine the significance threshold.

Asreml [16] was used for heritability estimation. And the kinship matrix was calculated using GEMMA (v0.98) [17] with the ‘-gk 1’ parameter. Subsequently, association analysis was performed using GEMMA’s univariate linear mixed model. The specific mixed model is as follows:

*y* = *Wα* + *xβ* + *u* + *ϵ*, (1)

where *y* is the phenotype vector; *W* is the covariate matrix (including sex, batch, and the first 5 PCs); *α* is a vector of corresponding coefficients (including the intercept term); *x* is the SNP genotype vector; *β* is the vector of SNP additive effects; *u* is the random effect vector; and *ϵ* is the residual. The most significant SNPs (lead SNPs) identified by GWAS were annotated using SnpEff (4.3) [18].

### 2.3. Post GWAS

To examine if the lead SNP polymorphism affects transcription factor binding, we extracted the sequence 30 bp around the lead SNP and used Alibaba2 [19] for transcription factor binding site (TFBS) prediction.

We also performed fine-mapping to identify the causal variants within a 300 kb range of the lead SNP, given the evidence of the significant association of the lead SNP in GWAS. We used SusieR [20] to estimate the posterior inclusion probability (PIP) of each SNP in the selected region, which indicates the evidence for SNP having a non-zero effect (namely, causal). We then ranked the SNPs by PIP and summed these until the cumulative sum reached 95%. All included SNPs are considered the credible set, which refers to the minimum set of variants that contains all causal SNPs with high probability.

For the SNPs in credible sets, we performed haplotype analysis using Haploview [21] and visualized the results with LDBlockShow [22]. The ggstatsplot R package [23] was used to analyze the association between haplotypes and phenotypes with default parameters.

## 3. Results and Discussion

### 3.1. Phenotypes and Heritability

Table 1 shows heritability and phenotype statistics for all individuals. BL averages 26.49 cm, KBL averages 13.58 cm, NL averages 21.06 cm and BrW averages 12.00 cm. FBL and NL are very similar to our previous findings, while BrW increased by approximately 1 cm [7].

The heritability estimations for BL (0.45) and BrW (0.40) suggest that nearly half of the phenotypic variation in body length and breast width can be attributed to genetic factors. BL is consistent with previous studies on similar poultry breeds, which reported heritability reached 0.5 in cross-bred chickens [24], while BrW is slightly higher than our previous results, where the heritability was 0.29 [7]. The heritabilities for KBL (0.30) and NL (0.37) also indicate a significant genetic influence, although slightly lower than for BL and BrW. The heritabilities of KBL and NL are lower compared to our previous research using GBS. This difference is probably due to the limitations of GBS, such as uneven genome coverage, which can lead to reduced sequencing accuracy in certain regions [25]. The relatively high heritability of these traits suggests that selective breeding could effectively improve these phenotypes in Pekin ducks. However, there are limitations to this assessment, such as the specific population and environmental conditions studied. Future research should aim to confirm these findings in larger and more diverse populations, and to investigate the potential effects of gene–environment interactions on these traits.

### 3.2. GWAS and TFBS Prediction

**Figure 1:**
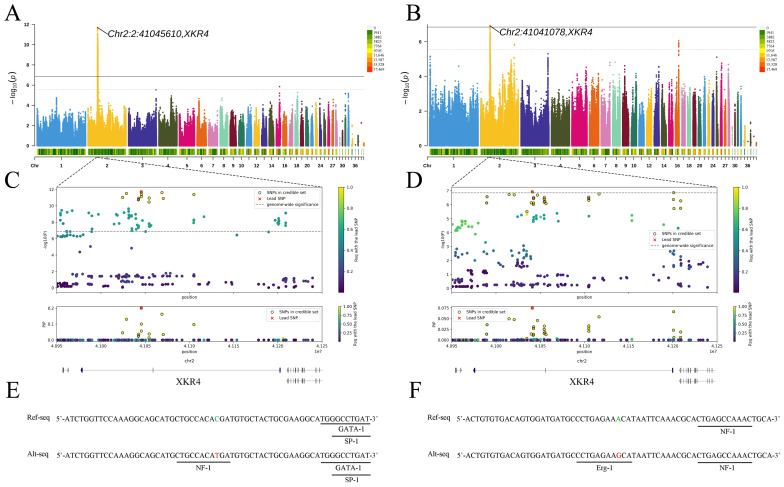
GWAS and post-GWAS analysis. The left side of the image represents body length (BL), and the right side represents keel bone length (KBL). (**A**,**B**) Manhattan plots of BL and KBL, respectively. the solid lines represent genome-wide significance level threshold lines (*p* = 1.40 × 10^−7^), and the dashed line represents the potential significance level threshold line (*p* = 2.82 × 10^−6^). (**C**,**D**) Fine-mapping results of BL and KBL, respectively. The red points represent the lead SNPs, and the points with black outlines represent the SNPs in credible sets. (**E**,**F**) Results of TFBS prediction for BL and KBL, respectively. The green bases indicate the reference genotype, while the red bases indicate the mutant genotype.

The GWAS results indicate that BL, KBL, and BrW show a significant signal on chromosome 2, while the significant signal for NL is located on chromosome 23. The details of the lead SNPs are shown in Table 2, and we found that the lead SNPs of BL and KBL are very close in position, differing by only 4.5 kb (Figure 1A,B). Furthermore, the allele frequencies of these SNPs are close to 0.5 in the population, suggesting that these alleles are not under strong artificial selection [26,27] and could be candidate genetic markers for selection in body size.

Further annotation using SnpEff revealed that the lead SNP of NL is located in the intergenic region, whereas the lead SNP for BrW is located within a pseudogene. However, both lead SNPs for BL and KBL are located within the intron of *XKR4*, close to the second exon. We hypothesize that these lead SNPs for BL and KBL may influence the size of Pekin ducks by affecting *XKR4*. *XKR4* (XK, Kell blood group complex subunit-related family, member 4) functions as a phospholipid-scrambling protein [28] and may play an important role in regulating the body size and growth of Pekin ducks. Previous studies have also extensively reported the association of *XKR4* with animal growth. Terakado et al. identified a significant association between the *XKR4* and birth weight in Nelore cattle using GWAS [29]. An et al. identified the *XKR4* as a candidate gene associated with body height and hip height in Chinese Wagyu beef cattle [30]. Lindholm-Perry et al. identified *XKR4* as a key candidate gene associated with growth traits in cattle [31]. These studies all point to *XKR4* as an important gene influencing growth and skeletal development in animals, providing support for our research findings.

The significant signals for BL and KBL are located in the intron of *XKR4*, suggesting that variation in these regions may regulate height traits by affecting gene expression or function. Previous research has shown that causal variants in *XKR4* may lie outside the coding region [32]. In addition, Yong et al. found that polymorphisms within the intron of the *XKR4* gene significantly affected body length, weight, thoracic circumference, and height in Boza goats [33]. Therefore, we used Alibaba2 to predict TFBS in the vicinity of the lead SNPs. The results show that in BL, the mutant allele of the lead SNP creates a binding site for the transcription factor NF-1 (Figure 1E). NF-1 is a widely distributed transcriptional regulatory protein in eukaryotes that plays a crucial role in the regulation of gene expression, cell differentiation, development, and other biological processes. It has been reported that NF-1 can bind to an enhancer element upstream of the mouse growth hormone receptor gene and positively regulate its transcription [34]. Similarly, the lead SNP mutation in KBL creates a binding site for the transcription factor Erg-1 (Figure 1F). Erg-1 is a zinc finger transcription factor that promotes osteoblast differentiation and bone formation by regulating the expression of skeletal-related genes [35,36]. Therefore, the occurrence of mutant alleles of the lead SNPs in BL and KBL may affect gene expression through similar mechanisms, further regulating body size in Pekin ducks.

### 3.3. Fine-Mapping and Haplotype Analysis

**Figure 2:**
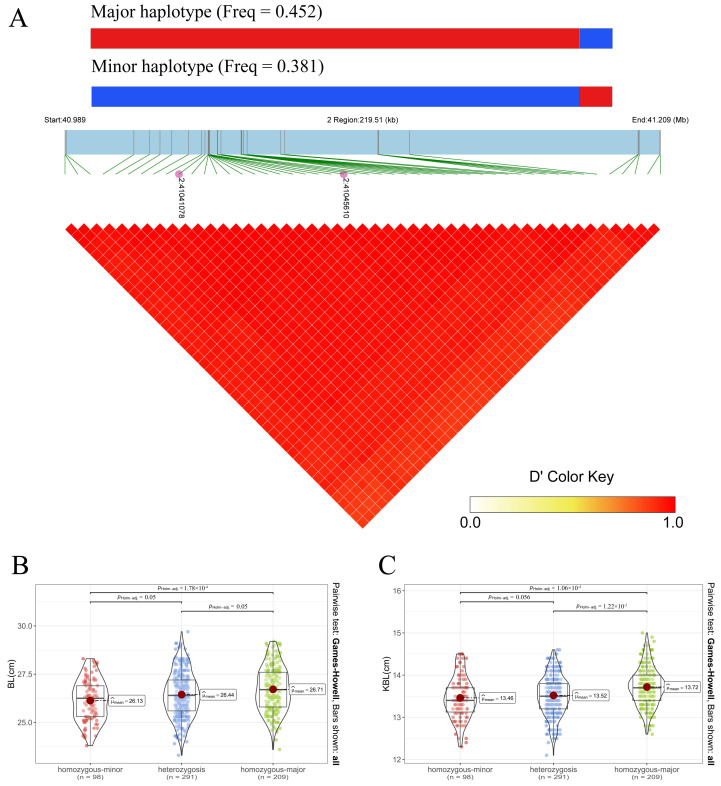
Linkage disequilibrium and haplotype analysis. (**A**) The haplotype frequency and linkage disequilibrium block of all 48 SNPs. (**B**,**C**) The correlation analysis of BL and KBL, respectively. Homozygous-minor represents individuals homozygous for the minor haplotype; heterozygosis represents individuals heterozygous for the minor and major haplotypes; homozygous-major represents individuals homozygous for the major haplotype; n represents the number of individuals.

To identify the causal variants for BL and KBL, we performed a fine-mapping analysis (Figure 1C,D). The credible sets for BL contained 13 SNPs, while for KBL, there were 35 SNPs. These SNPs represent the smallest sets containing the causal variants. Given their close positions, we combined all SNPs from the credible sets of both traits for linkage disequilibrium and haplotype analyses (Figure 2A). The results showed that these 48 SNPs were in high linkage disequilibrium. Two major haplotypes were present in our study population: the major haplotype with a frequency of 0.452 and the minor haplotype with a frequency of 0.381. The first 45 SNPs of the major haplotype showed the mutant genotype (red), while the last 3 SNPs matched the reference genotype (blue). Further association analysis between haplotypes and phenotypes (Figure 2B,C) showed that the major haplotype significantly increased both BL and KBL. Specifically, individuals homozygous for the major haplotype had an increase of 0.58 cm in BL and 0.26 cm in KBL compared to those homozygous for the minor haplotype. This supports our hypothesis that the lead SNPs cause changes in TFBS.

Explaining phenotypic variation in animals is complex because individual SNPs typically serve as weak instrumental variables for explaining phenotypic differences. However, the genome contains many linked variants, and using haplotypes as instrumental variables can strengthen the association between phenotypes and genetic variation, thereby increasing the reliability of causal inference [37]. Based on the lead SNPs, we identified a haplotype that significantly increased BL and KBL. The identification of this haplotype strengthens the phenotype–genotype association and improves the reliability of causal inference, suggesting that this haplotype is likely to be the causal haplotype responsible for the increase in BL and KBL.

## 4. Conclusions

This study identified QTL and candidate genes associated with body size traits in Pekin ducks. The results showed that most of the body size-related QTLs were distributed in intergenic and regulatory regions. More importantly, we identified two haplotypes within the *XKR4* in Pekin ducks, comprising 48 SNPs. These two haplotypes represent the major variants with a combined frequency of more than 80% and are likely to affect gene regulation by altering transcription factor binding sites. Additionally, we found that the proportion of mutant haplotypes in the population was almost equal to that of wild haplotypes, suggesting that this locus could be a potential candidate marker for future improvement of duck body size.

These findings not only contribute to a better understanding of the genetic basis of body size traits in Pekin ducks but also provide new insights and research strategies for exploring related gene regulatory networks. However, the specific role of these candidate genes or mutations in affecting changes in body size needs to be verified by molecular experiments. Additionally, further investigation into the performance of these haplotypes in different duck breeds and growth stages is necessary to fully understand their role in body size regulation.

## Figures and Tables

**Table 1:** Heritability estimation and phenotypic statistics.

Traits	(h^2^) ^5^	Means	Min	Max	CV ^6^	N ^7^
BL (cm) ^1^	0.45 ± 0.09	26.49 ± 1.16	23.30	29.70	4.38	641
KBL (cm) ^2^	0.31 ± 0.08	13.58 ± 0.47	12.10	15.00	3.48	641
NL (cm) ^3^	0.37 ± 0.08	21.06 ± 0.94	18.70	23.90	4.47	641
BrW (cm) ^4^	0.40 ± 0.09	12.00 ± 0.41	10.80	13.20	3.46	642

Note: ^1^: BL, body length; ^2^: KBL, keel bone length; ^3^: NL, neck length; ^4^: BrW, breast width; ^5^: h^2^, heritability; ^6^: CV, coefficient of variation; ^7^: N, the number of individuals.

**Table 2:** Identification of lead SNPs associated with BL and KBL.

Traits	Lead SNP	*p*-Value	Ref ^1^	Alt ^2^	MAF ^3^	Gene	Consequence ^4^
BL	2:41045610	2.02 × 10^−12^	C	T	0.457	*XKR4*	Intron variant
KBL	2:41041078	1.21 × 10^−7^	A	G	0.417	*XKR4*	Intron variant
NL	23:6102898	5.24 × 10^−7^	G	A	0.441	*-*	Intergenic variant
BrW	2:40713142	4.00 × 10^−7^	G	A	0.452	Pseudogene	Downstream variant

Note: ^1^: Ref, reference genotype; ^2^: Alt, mutant genotype; ^3^: MAF, minor allele frequency; ^4^: Consequence, the variant type.

## Data Availability

The data used in this study were deposited in the figshare repository (https://doi.org/10.6084/m9.figshare.26056162.v1).
